# A Health-Related Digital Ecological Momentary Assessment in Children (Aged 5– 11 Years): Systematic Review

**DOI:** 10.2196/79291

**Published:** 2026-04-14

**Authors:** Sydney Charitos, Lauren Thompson, Amberly Brigden, Jon Bird

**Affiliations:** 1 School of Engineering Mathematics and Technology, University of Bristol, 1 Cathedral Square, Bristol, BS1 5DD, United Kingdom, 44 117 42 82343 ext 82343

**Keywords:** children, systematic review, ecological momentary assessment, acceptability, feasibility, PRISMA, mobile phone

## Abstract

**Background:**

Digital ecological momentary assessment (EMA) collects data on experiences as they occur in daily life, capturing dynamic, context-sensitive experiences often missed by retrospective reporting. While EMA shows promise for pediatric health research, preadolescents have distinct socioemotional and cognitive characteristics likely to affect engagement. Existing reviews have not focused on the acceptability and feasibility of EMA protocols for this age group.

**Objective:**

This review aimed to examine digital EMA protocols used with children aged 5‐11 years across health domains, focusing on protocol characteristics, acceptability, and feasibility. We address 3 research questions (RQs)—RQ1: What are the characteristics of these protocols? RQ2: What is the feasibility and acceptability of these protocols? RQ3: What are the characteristics of high and low response rate protocols?

**Methods:**

We searched 10 databases (CINAHL, Embase, ACM Digital Library, IEEE Xplore, Cochrane Library, PsycINFO, Web of Science, PubMed, Scopus, and MEDLINE) for peer-reviewed studies published up to October 2025. Eligible studies used EMA with children aged 5‐11 years to collect health data via digital devices. Two researchers independently screened studies (SC and LT); one (SC) conducted quality assessment and data extraction. Findings were narratively synthesized.

**Results:**

We identified 17 protocols across 37 studies. Most targeted nonclinical populations, used handheld devices, spanned 3‐28 days, and applied interval-contingent prompting (RQ1). Response rates were available or calculable for 15 of 17 protocols, ranging from 48% to 92% (RQ2). Six protocols reported response rates of ≥80%. However, key data required for pooling (eg, raw counts for planned vs completed prompts) were missing or selectively reported. This contributed to 13 of 17 protocols being rated at critical risk of bias (ROBINS-I, v2). As a result, the strength of evidence was limited by poor reporting and high risk of bias. Facilitators included uncomplicated, engaging technology, reminders, and caregiver involvement. Barriers included device burden, restricted device access, difficulty with accurate reporting, stigma, limited device awareness, and insufficient caregiver support. High-response protocols (≥80%) often involved older children or clinical groups, ≥3-week duration, fixed schedules (≥20 items per prompt, 3 or 4 times per day), timing customization, and incentives (RQ3).

**Conclusions:**

This review provides the first systematic synthesis on preadolescents, offering insight into EMA protocol design beyond prior work treating children as a single group. By examining 17 EMA protocols, the review identifies gaps in developmental appropriateness and reporting quality, highlighting where the evidence may differ from adolescent and adult EMA research. The results suggest that digital EMA for preadolescents requires greater focus on child-centered design to increase acceptability and adherence, alongside improved reporting standards, so protocols can be meaningfully compared. With these advances, EMA could be more effectively integrated into pediatric health monitoring, tailored to the needs of different age groups.

## Introduction

### Background

Ecological momentary assessment (EMA) is a research method used to collect data on individuals’ behaviors, experiences, and physiological states as they occur in real time and in everyday settings [1]. EMA has proven particularly valuable in health research because it captures experiences that are often dynamic, personal, and shaped by context [2-4]. For example, symptoms such as pain, fatigue, or mood often fluctuate over short periods and may be influenced by daily routines, social interactions, or environmental triggers [5-7]. In contrast, traditional methods, such as retrospective reporting, may struggle to measure these experiences accurately due to recall bias and the influence of recency effects and emotional salience [1]. In recent years, the increasing availability of smartphones and wearable devices has supported the integration of EMA into individuals’ daily routines [8-10]. A typical EMA protocol uses these devices to deliver prompts, brief surveys sent to participants at scheduled times. Each prompt includes 1 or more questions, referred to as the item count, which can vary depending on the protocol’s aims and design [1].

EMA may be a particularly useful method for capturing health-related experiences in preadolescent children (aged 5‐11 years). Children at this age demonstrate metacognitive ability, reflected in their capacity to consider their own emotions and experiences, at a level similar to that of adults [11]. In contrast, traditional retrospective self-report approaches can be especially challenging for this age group [12]. At this developmental stage, children’s long-term memory is still maturing, which can limit how accurately they recall past events [13]. They can also find it difficult to verbally express their health experiences, especially in unfamiliar clinical settings or when speaking with health care professionals they do not know personally [14]. EMA may help overcome these barriers by enabling in-the-moment reporting rather than requiring reflection on past experiences.

However, a central challenge in implementing an EMA protocol is achieving both acceptability, that is, how suitable, engaging, or satisfying the method is perceived to be by participants, and feasibility, that is, how practical and realistic it is to implement [15]. These dimensions are essential for ensuring sustained participation and the collection of high-quality data. As a result, researchers have explored a variety of methodological adaptations aimed at improving protocol acceptability and feasibility. For example, micro-EMA is a protocol design that uses ultrabrief prompts (within a few seconds) to reduce participant burden [16]. Researchers have also incorporated context-aware delivery methods that adjust prompts based on user behavior or timing to minimize disruption [17,18]. Such adaptations reflect the ongoing evolution of EMA as researchers refine approaches to better support acceptability and feasibility across a range of participants and contexts.

EMA has consistently demonstrated acceptability and feasibility in adult populations, with average response rates (the proportion of scheduled prompts completed by participants) frequently exceeding 80% [19], a standard of high response rate in EMA studies [20]. Although certain protocol features, such as item count and prompt frequency, can influence response rates, adults generally maintain strong response levels (above 70%) despite these variations [19]. A factorial experiment designed to isolate the individual effects of specific protocol characteristics found that common variations did not significantly affect response rates [21]. Instead, individual differences and the interactions between protocol elements were identified as having a more substantial influence on response rates. These findings indicate that EMA protocols for adult populations can be flexibly adapted to specific research requirements without substantially compromising response rates.

Compared with adults, existing evidence on response rates in EMA protocols for preadolescent children remains limited and inconclusive. A 2023 meta-analysis suggested that age may not be a strong predictor of EMA response rate; however, this analysis included only 6 protocols involving children younger than 12 years (1.2% total sample), limiting confidence in the finding for this age group [22]. Furthermore, many reviews aggregate data across preadolescent (aged 5‐11 years) and adolescent (12‐18 years) age groups, making it difficult to isolate trends specific to younger children [23-25]. For example, Wen et al [23] examined mobile EMA with “children and adolescents,” but only one of their included studies overlaps with the present review, illustrating how younger children are rarely the focus of EMA research. Their review focused on mobile EMA in health science databases and largely adolescent samples, whereas the present review searched a wider range of interdisciplinary sources, including Human-Computer Interaction (HCI) venues, and targeted protocols specifically designed for preadolescent children (aged 5‐11 years). Within this aggregation, there is some evidence that children and adolescents may exhibit lower response rates than adults; a systematic review reported an average compliance rate of 78.3% among pediatric samples, below the levels typically observed in adult EMA studies [23]. Despite this tendency to group age bands or assume similar patterns across age groups, systematic reviews nonetheless emphasize the importance of adapting EMA protocols to better meet the developmental and contextual needs of younger children [23,24,26]. Recommendations include restricting prompts to occur outside school hours and limiting internet access to support caregiver oversight [24]. While such recommendations are noted within existing systematic reviews, they are not typically the primary focus, underscoring the need for more targeted research on how EMA protocols can be effectively adapted for younger children.

### Aims

This systematic review aimed to investigate how EMA protocols are being used with children aged 5‐11 years, across health domains and population characteristics, focusing on their acceptability and feasibility for children. This review aimed to build on the established literature on the adherence of children to EMA protocols by investigating the acceptability and feasibility of these different protocol characteristics and understanding how current protocols attempt to improve adherence. In this review, we investigated the following research questions (RQs): (1) What are the characteristics of health-related EMA protocols being used with children aged 5-11 years? (2) What is the feasibility and acceptability of these EMA protocols with children aged 5-11 years? (3) What characteristics of EMA protocols, RQ1, are related to high and low response rate, RQ2, when using EMA with children between the ages of 5 and 11 years?

## Methods

This review was registered in the PROSPERO database and follows the guidance of the PRISMA (Preferred Reporting Items for Systematic Reviews and Meta-Analyses) guidelines [27]. See PRISMA and PRISMA-S checklists.

### Search Strategy

The literature search was first conducted on March 13, 2023, and updated on October 28, 2024, and October 15, 2025, in the following databases: CINAHL, ACM Digital Library, PsycINFO, Embase, MEDLINE, Cochrane Library, IEEE Xplore, PubMed, Scopus, and Web of Science. Each database was searched via its native platform (eg, MEDLINE via Ovid, PsycINFO via Ovid, and Scopus via Elsevier), with full platform—database mappings and exact search strings provided in PRISMA-S checklist. These were chosen to span multiple disciplines including medical and HCI research. The search included key terms for (1) children aged 5-11 years, such as “school child” or “minor” and (2) EMA, such as “Experience Sampling” or “Ambulatory Assessment” (Multimedia Appendix 1 [28,29] provides the full list of search terms used). No other limits or restrictions were placed on searches other than date restrictions for subsequent literature reviews in 2024 and 2025. The search strategy was not formally peer reviewed but was discussed with the wider research team. No study registries, conference proceedings, organizational websites, or gray literature sources were searched, as the review focused on peer-reviewed empirical studies. The search and reporting methods were documented in accordance with the PRISMA-S (PRISMA Search) checklist, with full database-specific search strategies, platforms, and adaptations reported in PRISMA-S checklist.

### Inclusion and Exclusion Criteria

Studies were included if they fulfilled the following criteria: (1) peer-reviewed or empirical studies with any study design, (2) published in English, (3) the research involved the use of EMA to collect health-related information (physical, mental, or social) on a child (aged 5‐11 years) either by the child themselves or proxy-reported by a parent or a caregiver, and (4) a digital technology (eg, smartphone) was used to collect EMA data. For the purpose of this review, EMA is defined as the assessment of a phenomenon in natural settings at least twice a day [30]. Studies were excluded if they fulfilled the following criteria: (1) the study included children younger than 5 years or older than 11 years or did not report adaptations specific to the preadolescent age range (5‐11 years). Protocols designed for a broader group of children (eg, aged 5‐18 years) without age-specific considerations were excluded, as the focus of this review was on protocols intentionally tailored for preadolescents; or (2) the paper was considered a review on previously reported studies, protocol, nonempirical or nonscientific paper (eg, book chapters), or when the full text was not available.

### Screening Procedures

The search results were imported into Zotero (Corporation for Digital Scholarship) and duplicates were removed. In stage 1, each title and abstract was independently screened by 2 authors (SC and LT) for relevance. If an inclusion decision could not be made based on the title and abstract, the papers were included for full-text review. Papers deemed relevant at the title- and abstract-screening stage moved onto stage 2 where they were independently double-screened against the inclusion and exclusion criteria using the data management platform Rayaan (Rayyan Systems Inc), with reasons for exclusion recorded. Disagreements at both stages were discussed and resolved in meetings by the reviewers (SC and LT), with persistent disagreements being resolved through discussion with the wider research team (AB and JB). If the full text was not available through institutional subscriptions, interlibrary loan, or author contact (2 email attempts), the study was excluded. Where the full text was available but did not contain the information needed, 2 attempts were made to contact authors by email; if the information was not provided, the study was excluded. In addition, reference lists of included studies were manually screened to identify any additional eligible papers.

### Data Extraction and Synthesis

**Figure 1.**
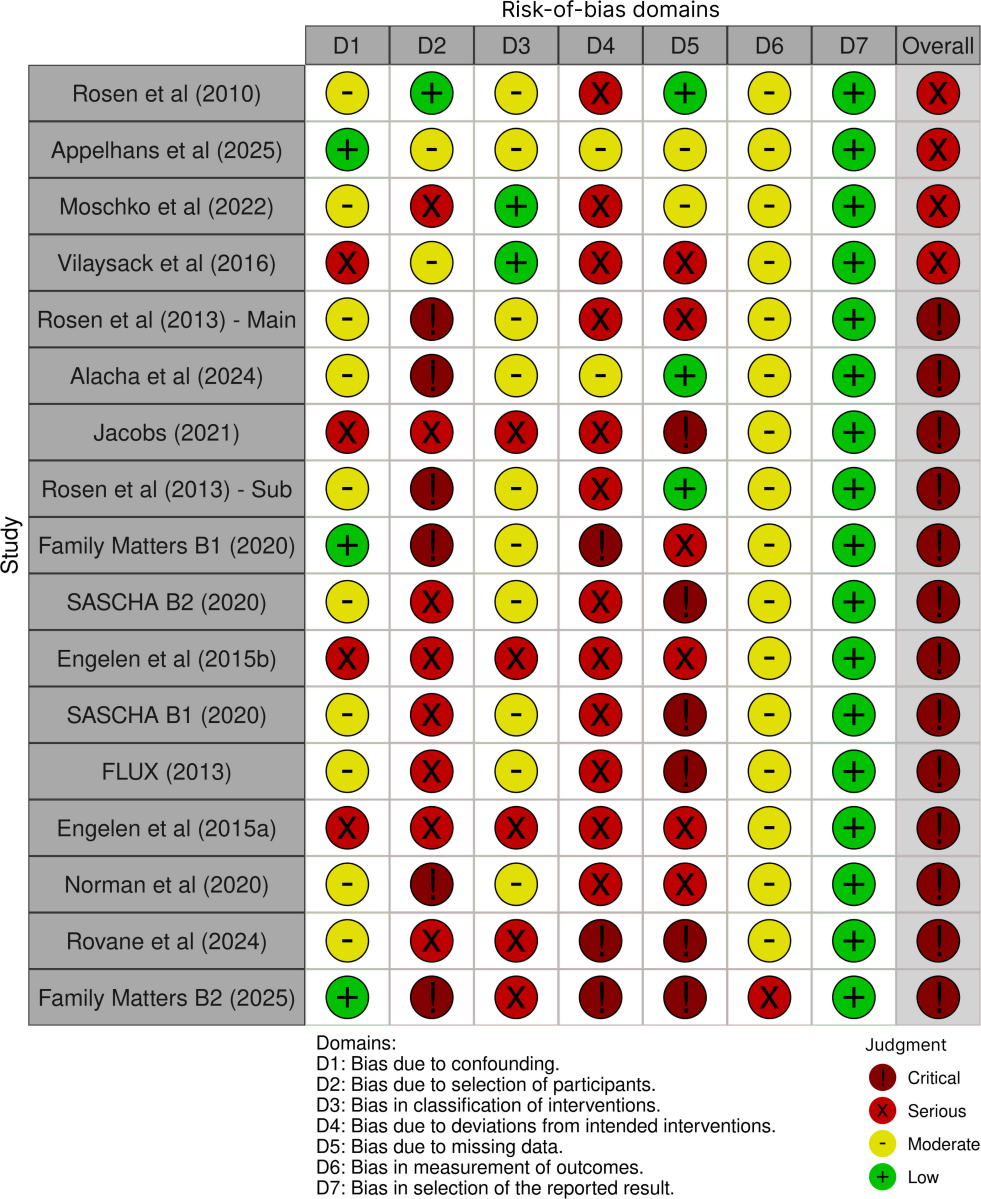
Summary of risk of bias for the 17 identified ecological momentary assessment protocols, assessed using the Risk Of Bias In Nonrandomized Studies of Interventions, version 2 (ROBINS-I v2) using robviz tool [33]. The figure displays domain-level and overall ratings, showing that most protocols (13 out of 17) were judged to be at critical risk of bias [31-60,62].

Data were extracted by the primary author (SC), following the protocol in Textbox 1, which aligns with the Checklist for Reporting EMA Studies (CREMAS) [25]. In line with our PROSPERO registration, methods were adapted during analysis: RQ3 was added to examine protocol features linked to response rates, while planned technology-based taxonomies were not applied due to insufficient reporting. A quantitative meta-analysis was not feasible, as key data required for pooling (eg, raw counts for planned vs completed prompts and corresponding variance estimates) were missing or selectively reported across protocols. This missingness contributed to 13 of 17 protocols being judged at critical risk of bias during quality assessment (see Multimedia Appendix 2 [31-60] for full assessments and Figure 1 for a summary). As a result, a narrative synthesis was undertaken [61]. Pooled response rate estimates were calculated for the subset of protocols not judged at critical risk of bias using a random-effects model, with mean response rates, 95% CIs, and a prediction interval reported.

The search identified 31 eligible papers describing 37 studies. As several papers reported on the same underlying methodology, and some studies included distinct subgroups with methodological differences, these were consolidated into 17 unique EMA protocols, which formed the unit of analysis for this review. Where protocols spanned multiple papers, these were grouped under project or funding names, with “main” or “sub” labels applied where necessary. Protocols were named based on the explicit project name when provided or if this was not available, the funding body.

Textbox 1. **Data extraction categories and details extracted.**

General protocol aim and participants (study-level information included in Multimedia Appendix 3 [31-60]).

- Publication details: authors, year of publication, country of publication, and publication location
- Sample size
- Demographics: participant age, health condition if specified
- Protocol aim

Research question (RQ) 1: What are the characteristics of the ecological momentary assessment (EMA) protocol?

- EMA purpose (eg, intervention or observational tool)
- Training (included in the Checklist for Reporting EMA Studies [CREMAS]): child and parent
- Technology (included in the CREMAS): reporter, device, software, response method, any additional devices (eg, accelerometer)
- Monitoring period (included in the CREMAS)
- Prompting design (included in the CREMAS): strategy (eg, interval, event-contingent), number of questions
- Prompting frequency (included in the CREMAS)
- Design features (included in the CREMAS): piloting (item and protocol level), reward for participation, customization, reminder systems, support networks, and protocol flexibility.

RQ 2: Is it feasible and acceptable to use digital devices to implement an EMA methodology with children between the ages of 5 and 11 years for health purposes?

- Feasibility details: dropout, attrition, response latency, and response rate (planned vs complete).
- Acceptability details: qualitative evidence relating to acceptability and feasibility (eg, exit interviews)
- Any information captured about acceptability and feasibility but not captured through formal qualitative or quantitative data collection, that is, authors’ interpretations in the results and discussion.

RQ 3: What characteristics of EMA protocols, RQ1, are related to high and low adherence, RQ2, when using EMA with children between the ages of 5 and 11 years?

- Additional synthesis of data extracted from RQ1 and RQ2, see “Data Extraction and Synthesis” section for details.

For RQ1, we extracted the frequency of each protocol feature and synthesized findings through descriptive statistics. To ensure consistency, ranges were reported when no overall metric was provided across subanalyses. Within the CREMAS design features category, 5 subcategories were identified to support consistent reporting: rewards for participation, piloting, customization, reminder systems, and support networks.

For RQ2, we extracted adherence-related metrics (including response rate, attrition, and latency) and qualitative data on acceptability and feasibility. If overall response rates were not reported, they were calculated from raw data (planned vs actual responses). Full calculations can be found in Multimedia Appendix 4 [31-60]. The planned metaethnography was not feasible, as only 2 of 17 protocols included formal qualitative research on acceptability and feasibility. Vilaysack et al [31] provided a summary of feedback; Norman et al [32] applied manifest content analysis to identify barriers (demanding, challenging in irregular situations) and facilitators (uncomplicated, engaging). Instead, we applied a thematic synthesis approach [63], using the framework of barriers and facilitators by Norman et al as a deductive starting point and expanding it inductively to incorporate additional author observations and participant quotes.

For RQ3, which examined how protocol characteristics (RQ1) relate to response rate patterns (RQ2), no additional data were extracted. Instead, to synthesize data for this RQ, protocols were categorized as high or low response rate using an 80% threshold (Stone and Shiffman [20]). Researchers then identified protocol characteristics (identified in RQ2) found in >50% of protocols within each group.

### Quality Assessment

One author conducted a quality assessment, discussing any borderline assessments with the wider team. We used 2 complementary tools: the CREMAS [25], and the Risk Of Bias In Nonrandomized Studies of Interventions, version 2 (ROBINS-I v2) [64]. The CREMAS is a 16-item tool designed to enhance the transparency and rigor of EMA reporting. For the purposes of this review, each item was assessed using a binary coding scheme (“present” or “not present”), with ambiguous cases conservatively marked as “not present.” The ROBINS-I v2 tool was used to evaluate risk of bias across 7 domains. Full details of both assessments are provided in Multimedia Appendix 2, with a summary of ROBINS-I v2 ratings shown in Figure 1.

## Results

**Figure 2.**
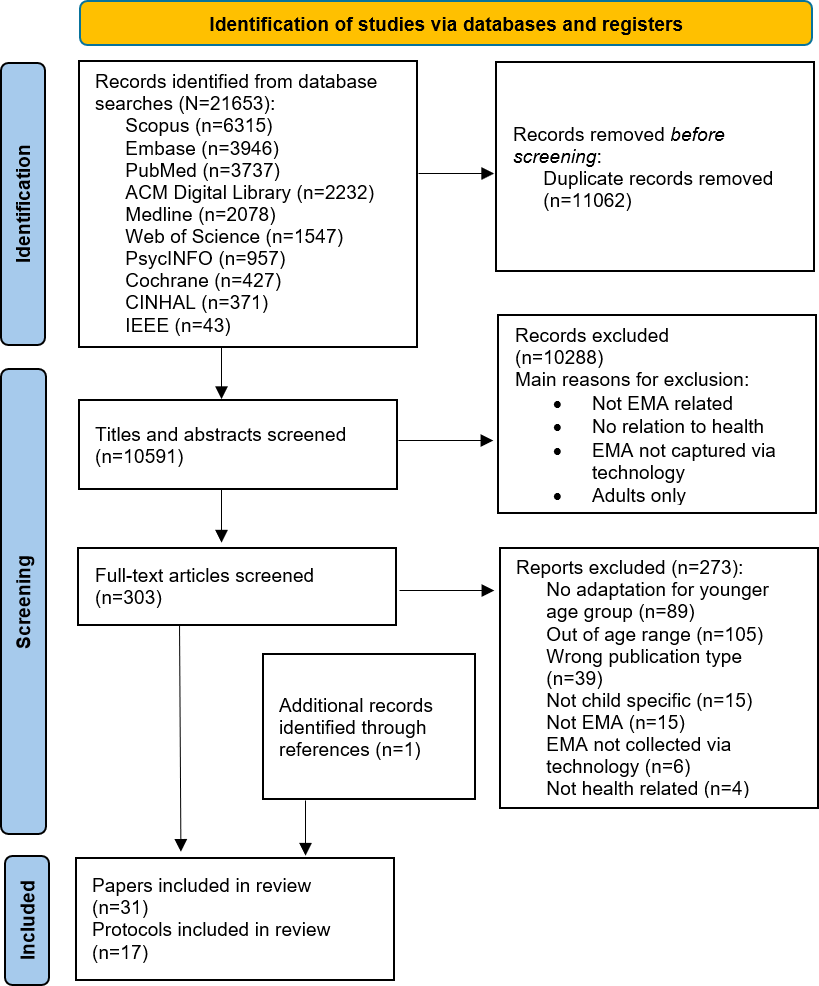
PRISMA (Preferred Reporting Items for Systematic Reviews and Meta-Analyses) flow diagram. EMA: ecological momentary assessment.

The PRISMA flowchart is represented in Figure 2. In this section, we synthesize data extracted to address our RQs as outlined in Textbox 1. Protocols are grouped by overall ROBINS-I rating (critical, serious, or moderate) and sorted within each group by response rate to facilitate comparison across quality levels.

### *Research question 1*: What are the characteristics of the EMA protocol?

For RQ1, Tables1^3^ summarize key characteristics of the included protocols. Table 1 summarizes protocol domains and participants; Table 2 outlines training and technology features; and Table 3 details prompt implementation.

**Table 1.** Overview of included ecological momentary assessment protocols, including study location, sample size, child age, condition, and primary aim (RQ1).

Protocol	Country	Sample size	Child’s age (years)	Condition	Aim
Serious					
Rosen and Epstein (2010) [33]	United States	2	8-11	ADHD^a^	To assess links between parental stress, feeding practices, and child-eating behaviors via EMA^b^.
Appelhans et al (2025) [34]	United States	60	5-10	None	To examine whether parent-supported recreational activities can displace discretionary eating and electronic entertainment.
Moschko et al (2022) [35]	Germany	70	9-11	ADHD	To investigate daily self-regulation in children with ADHD and parent-child interactions.
Vilaysack et al (2016) [31]	Australia	10	5-7	None	To assess EMA feasibility with typically developing children.
Critical					
Rosen et al (2013)—Main [36]	United States	11	7-11	ADHD	To examine parent EMA proxy reports of children’s emotional dysregulation.
Alacha et al (2024) [37]	United States	47	9-10	None	To investigate effects of positive affect variability on homework problems in children with ADHD.
Jacobs (2021) [38-40]	Germany	84	8-11	ADHD	To explore links between emotional regulation, sleep, and well-being using ambulatory methods.
Rosen et al (2013)—Sub [36]	United States	5	5-7	None	To explore how children can use EMA to self-report on their own emotional dysregulation.
Family Matters B1 (2020) [41-47]	United States	128-150	9-11	None	To examine diets of low-income, racially and ethnically diverse families.
SASCHA B2 (2020) [40,48-50,62]	Germany	108	5-7	None	To assess how transition to secondary school affects well-being and academic success.
Engelen, Bundy, Lau et al (2015) [51]	Australia	20	9-11	None	To examine links between children’s activity levels and contextual factors.
SASCHA B1 (2020) [48,50,62]	Germany	90	8-11	None	To investigate self-esteem, peer ties, and academic functioning before secondary school.
FLUX (2013) [38,52-57]	Germany	82-110	9-11	ADHD	To examine how sleep, activity, affect, peers, and worry relate to working memory and well-being.
Engelen, Bundy, Bauman et al (2015) [58]	Australia	246	5-7	None	To assess EMA feasibility for describing after-school activity patterns.
Norman et al (2020) [32]	Sweden	20	5-7	None	To examine feasibility and validity of photo-based EMA for children’s diets.
Rovane et al (2025) [59]	United States	92	5-11	ASD^c^	To explore links between parental emotion regulation, stress, and child behavior in ASD.
Family Matters B2 (2025) [60]	United States	436	5-7	None	To examine how parental stress, mood, and coping relate to children’s physical activity and screen time in daily life.

a ADHD: attention-deficit/hyperactivity disorder.

b EMA: ecological momentary assessment.

c ASD: autism spectrum disorder.

**Table 2.** Summary of protocol training and technology features across ecological momentary assessment protocols with children aged 5-11 years (research question 1).

Protocol	Reporter	Training child, parent	Technology	Response method	Reported question domain(s)
Serious					
Rosen and Epstein (2010) [33]	Parent	N^a^, Y^b^	PDA^c^	VAS^d^ (11: −5 to 5), VAS (10: 1 to 10), Categorical, and Likert (5)	Affect
Appelhans et al (2025) [34]	Parent	NR^e^, Y	Smartphone	Multi-Choice, Single-Choice	Activity (type), diet (food intake)
Moschko et al (2022) [35]	Child, parent	Y, NR	Child: smartphone; parent: survey (online or paper)	Likert (6)	Self-regulation, social relationships
Vilaysack et al (2016) [31]	Child	Y, Y	Smartphone	VAS (NR), Multi-Choice, Categorical	Activity
Critical					
Rosen et al (2013)—Main [36]	Parent	NR, Y	PDA	VAS (NR)	Affect
Alacha et al (2024) [37]	Child	NR, Y	Mobile phone	Likert (5)	Affect
Jacobs (2021) [38-40]	Child, parent	Y, NR	Smartphone	Likert (5), Working Memory Updating Task, and Time Picker	Sleep, affect, and working memory
Rosen et al (2013)—Sub [36]	Parent	NR, Y	PDA	VAS (NR)	Affect
Family Matters B1 (2020) [41-47]	Child	NR, Y	Tablet. optional: phone, accelerometer	Categorical, VAS (NR), Multi-Choice, and Likert (NR)	Food intake, stress, sleep, affect, and activity
SASCHA B2 (2020) [40,48-50,62]	Parent	Y, Y	Smartphone (app)	Likert (5), Working Memory Updating Task, and Time Picker	Sleep, affect, social relationships, working memory, self-esteem, achievement goals, self-regulation, and academic success
Engelen, Bundy, Lau et al (2015) [51]	Child	NR, NR	PDA, accelerometer	Multi-Choice, VAS (NR)	Activity
SASCHA B1 (2020) [48,50,62]	Child	Y, Y	Smartphone	Likert (5), Working Memory Updating Task, and Time Picker	Social relationships, affect, self-esteem, achievement goals, self-regulation, working memory, and academic success
FLUX (2013) [38,52-57]	Child, Parent	Y; Y	Smartphone, accelerometer	Likert (5), Working Memory Updating Task, and Time Picker	Affect, working memory, and sleep
Engelen, Bundy, Bauman et al (2015) [58]	Parent	NR, Y	PDA	Multi-Choice, VAS (NR)	Activity
Norman et al (2020) [32]	Child	NR, Y	Mobile phone	Photo, Free Text	Food intake
Rovane et al (2025) [59]	Parent	NR, Y	Smartphone	Likert (9), Categorical, and Likert (5)	Stress, behavior
Family Matters B2 (2025) [60]	Parent	NR, Y	Smartphone	Single-Choice, VAS (NR), Multi-Choice, and Likert (NR)	Diet, stress, sleep, affect, and activity

a N: Training did not happen.

b Y: Training is reported.

c PDA: personal digital assistant.

d VAS: visual analog scale.

e NR: not reported.

**Table 3.** Summary of prompt implementation details across ecological momentary assessment protocols with children aged 5-11 years (research question 1).

Protocol	Period	Strategy	Type^a^	Items, n	Frequency, n	Prompt interval
Serious						
Rosen and Epstein (2010) [33]	28 days	Interval	Fixed	29	3	Before school, after school, and evening
Appelhans et al (2025) [34]	17 days	Interval	Pseudorandom	4-5	3	Pseudorandom times during non–school hours and through entire day on weekends
Moschko et al (2022) [35]	18 days; 3× over 13 months	Interval	Random	7- 8	3 children; 1 parent	Before school, afternoon, and evening
Vilaysack et al (2016) [31]	7 days	Interval	Random	7	8	Random during waking hours (including school)
Critical						
Rosen et al (2013)—Main [36]	28 days	Interval	Fixed	1	3	Before school, after school, and evening
Alacha et al (2024) [37]	7 days	Interval	Fixed	20	3	Morning, afternoon or after school, and evening
Jacobs (2021) [38-40]	21 days	Interval	NR^b^	17-25	4	Before school (later on weekends), afternoon, and evening
Rosen et al (2013)—Sub [36]	28 days	Interval	Fixed	1 child; 2 parents	3	Before school, after school, and evening
Family Matters B1 (2020) [41-47]	8-10 days	Interval, event	Fixed, random	10-30	5 + event	Even split across waking hours
SASCHA B2 (2020) [40,48-50,62]	4 weeks	Interval	NR	28-41	4	Before school, morning (including school), afternoon, and evening
Engelen, Bundy, Lau et al (2015) [51]	4 days; 2× 13-week gap	Interval	Random	12	3	Between afternoon and evening
SASCHA B1 (2020) [48,50,62]	4 weeks	Interval	NR	28-41	4	Before school, morning (including school), afternoon, and evening.
FLUX (2013) [38,52-57]	4 weeks	Interval	Fixed	21-26	4	Morning (including school), midday (including school), afternoon, and evening
Engelen, Bundy, Bauman et al (2015) [58]	4 days; 2× 13-week gap	Interval	Random	12	3	Between afternoon and evening
Norman et al (2020) [32]	3 days	Interval, event	Fixed	1	1 + event	Evening
Rovane et al (2025) [59]	7 days	Interval	Random	3	5	Random during waking hours
Family Matters B2 (2025) [60]	7 days +	Interval	Fixed, random	10-34	4	Randomly within 3-hour window, EoD^c^ available later in the day

a Studies typically described prompting as “random” or “pseudorandom,” but few reported whether constraints (eg, minimum time between prompts) were applied. Our synthesis, therefore, reflects the terminology reported by authors, acknowledging that “random” may have been implemented differently across protocols.

b NR not reported.

c EoD: end of day.

#### General Protocol Domains and Participants

Table 1 summarizes protocol domains and participants (RQ1). Across the 17 EMA protocols, most (n=13/17) were designed to focus solely on either key stage (KS) 1 (ages 5‐7 years; n=5) or KS2 (ages 8‐11 years; n=8) [33,35,36,38-40,48-50,52-57,62] compared with KS1 [31,32,41-47,51,58]. Protocols sampled typically developing children [31,32,34,36,37,40-51,58,60,62] more frequently than those with specific conditions: attention-deficit/hyperactivity disorder (ADHD; n=5) [33,35-37] and autism spectrum disorder (n=1) [59]. Female representation varied (0%‐56%) but was between 40% and 56% in 13 protocols [31,32,34,35,37-58,60,62] and often lower among condition-related protocols, with 4 of 6 having ≤23% female participants [33,35,36,59].

*Research question 1*: What are the characteristics of the EMA protocol?

#### Reporter Training

Most protocols (n=15/17) used a single reporter: usually parent-reported in KS1 (n=7) [32,34,37,41-47,51,58-60], whereas protocols involving KS2 were split between child self-reports and parent proxy reports (n=6 child; n=7 parent) [35,36,38-40,48-50,52-57,62]. Where reported (n=5), parent respondents were predominantly female (74%‐100%) [32,33,35,41-47,59]. Child training was reported in 6 protocols and always involved verbal instruction with hands-on practice, with session durations ranging from 45 minutes to 4.5 hours when reported (n=3) [31,38-40,52-57]. Parent training was reported in 14 protocols [31-34,36,37,40-50,58-60,62]. Of these, 9 provided verbal instruction [31,33,34,37,40-50,59,60,62], 6 provided written materials [32,38,40-50,52-58,62], and 6 offered hands-on practice [31,33,34,41-47,59,60]. When reported (n=3), parent training sessions ranged from 15 to 45 minutes [31,33,59]. Three protocols provided training to both children and parents when the child was the reporter [31,40,48-50,52,53,55-57,62], but only 1 protocol included practice-based training for parents in this context [31]. In protocols where both child and parent were reporters (n=2), only 1 member of the pair was reported to have received training [35].

### Technology

Nearly all protocols (n=16/17) used dedicated handheld devices, most commonly smartphones (n=11) [31,32,34,35,37-40,48-50,52-57,59,60,62] or Personal Digital Assistants (n=5) [33,36,41-47,51,58], with 1 using a tablet [41-47]. Three protocols paired EMA with accelerometers for passive tracking [38,41-47,51-57]. Twelve protocols provided devices, typically restricting functionality to the research app (n=9) [33,35,36,38-50,55-57,62], while 5 allowed personal device use [32,34,35,37,60]; this occurred only when parents were reporting.

### Response Methods

The most popular response collection method was a Likert scale (n=10/17) [33,35,37-48,50,52-57,59,60,62], most commonly 5-point scale (n=7) [33,37-40,48-50,52-57,59,62]. Nearly all (n=14) combined 2 or more response types, often pairing Likert scales with visual analog scales or categorical options.

### EMA Purpose and Domain

All protocols used EMA as an observational tool and not an intervention. Twelve captured multiple domains, while 5 focused on a single domain [31,32,37,51,58]. Affect was most common (n=10), followed by activity (n=6) and sleep (n=5).

### Monitoring Period

Monitoring periods across the 17 protocols spanned from 3 to 28 days for a single wave: ≤7 days (n=7) [31,32,37,51,58-60], 8‐14 days (n=1) [41-47], 15‐21 days (n=3) [35,38-40], and ≥22 days (n=6) [33,36,38,40,48-50,52-57,62]. Only the Family Matters protocols (n=2) [41-47] included flexibility beyond their stated monitoring periods—extending participation if families did not complete a “full day” of EMA (2 of 4 interval‐contingent prompts, 1 mealtime survey, and 1 end‐of‐day survey).

### Prompt Design and Frequency

All protocols included interval-contingent prompting, typically 3-4 times per day (n=13/17) [33-40,48-58,60,62]. Prompt timing followed three main patterns: (1) 3 windows per day (before school, after school, and evening; n=5) [33,35-40], (2) 4 windows (adding a midmorning school prompt; n=4) [35,43-46,48-52], (3) or prompts spaced evenly or randomly across waking hours (n=5, one including schooltime prompts with child reporters) [31,34,41-47,59,60]. One protocol [32] added an end-of-day prompt mid-study after a poor response rate to daytime event-contingent prompts was observed. Six protocols varied item count by time of day (eg, shorter morning and longer end-of-day surveys) [35,38-50,52-57,62]. Response windows ranged from 3 minutes to 6 hours, with 2 protocols linking window length to the interval until the next prompt [40,48-50,62] and 2 adjusted it by time of day [41-47,60].

### Design Features

Design features relevant to reporting bias and participant burden were categorized into 5 domains—rewards, piloting, customization, reminders, and support. These domains are summarized, with detailed protocol-level information provided in Multimedia Appendix 5 [31-60,62].

#### Rewards for Participation

Of the 17 protocols, 13 offered participant reimbursement. Fixed payments, meaning amounts provided regardless of response rate, were reported in 5 protocols [35,37-40,48-50,59,62]. Six protocols tied bonuses to response rate thresholds of 60%‐90%, typically offering an extra US $6 to US $12 for meeting these targets (n=3) [38-40,48-50,59,62], or stating that the total payment was dependent on response rate (eg, up to US $100) (n=5) [34,38,41-48,50,52-57,60,62]. The maximum payment per prompt ranged from US $0.48 [37] to US $2.68 [60], with an average of US $0.95 per prompt. Six protocols supplemented cash with nonmonetary incentives (eg, iPad [41-47], activity center tickets [32], and data summary [59]). Two protocols explicitly offered no reimbursement [36].

#### Piloting

Only 2 of the 17 protocols reported fully piloting its EMA protocol [31,60]. In terms of item piloting, 3 protocols reported piloting individual items with children from the target age group [38-40,52-57]. Three protocols adapted existing non-EMA measures for some, but not all, EMA items, with limited detail on sources or testing [32,35,40-50,54,55,61,62].

#### Customization to Protocol

Of the 17 protocols, 11 allowed some customization to support adherence, which always involved tailoring prompt timing to participants’ daily routines at the start of the study, with 1 protocol providing the option to update this midway through the study period [33]. Only 2 protocols offered multiple customization options including delivery method (text or email) and EMA question language [41-47], or letting parents choose between paper and digital surveys [35].

#### Reminder Systems

Four protocols explicitly referenced reminder systems [31,32,41-47,60]. To prompt event-contingent entries, 1 protocol used scheduled text reminders [32] and another embedded the event-contingent survey at the start of the interval-contingent prompt (n=1) [41-47]. One protocol triggered follow-up contact after 2 days of missed data [41-47]. Another adjusted prompt volume to minimize disruption—silent in the morning, vibrating during school hours, and audible in the evening [48,50,62]. Additional strategies included follow-up reminders after the main prompt [31,60], sticker tracking sheets [41-47], and regular caregiver check-ins (every 2-3 days) [31].

#### Support Networks

Eight protocols reported support systems via 3 main groups. Research staff often assisted (eg, hotline, in-person meeting, and scheduled calls) (n=5) [33,34,41-48,50,60,62]. Teaching staff or schools contributed to 4 protocols, more passively by allowing the protocol to occur [38-40,48-50,62] and more actively by monitoring survey completion and response times [38,52-57]. Parents were only formally integrated in an explicit support role in 1 protocol [52,55-57], where they were asked to track their child’s EMA completion.

*Research question 2*: Is it feasible and acceptable to use digital devices to implement an EMA methodology with children between the ages of 5 and 11 years for health purposes?

### *Research question 2*: Is it feasible and acceptable to use digital devices to implement an Ecological Momentary Assessment methodology with children between the ages of 5 and 11 for health purposes?

In total, 11 of the 17 included protocols did not report the overall response rate across all question domains [32-35,43-53,55,62,63]. As a result, the values in Table 4 were calculated from available data, where possible; further details on these calculations can be found in Multimedia Appendix 1.

**Table 4.** Summary of feasibility and acceptability outcomes across ecological momentary assessment protocols with children aged 5-11 years (research question 2).

Protocol	Dropout: reason	Exclusion from analysis: criteria	Technical issues	Average response latency in minutes (range)^a^	Child RR^b^	Parent RR
Serious						
Rosen and Epstein (2010) [33]	0%: NR^c^	0%: NR	NR	NR	N/A^d^	91%^e^
Appelhans et al (2025) [34]	NR	NR	NR	NR	N/A	70%
Moschko et al (2022) [35]	30%^f^ (between waves): NR	4%: No parent data	3%	5	62%^e^	56%^e^
Vilaysack et al (2016) [31]	NR	NR	NR	1.75 (0.5-3)	48%^e^	N/A
Critical						
Rosen et al (2013)—Main [36]	NR	13%^f^: NR	13%	NR	N/A	87%
Alacha et al (2024) [37]	NR	36%^f^: <5 successive ratings	NR	NR	N/A	86%
Jacobs (2021) [38-40]	NR	NR	NR	6.5 (3-10)	85%^e^	N/A
Rosen et al (2013)—Sub [36]	NR	NR	NR	NR	84%	NR
Family Matters B1 (2020) [41-47]	NR	NR; incomplete survey set. 4%-6% (accelerometer: <4 days and <4 hours per day)	NR	3.5 (2-5)	N/A	80%
SASCHA B2 (2020) [40,48-50,62]	NR	NR	NR	NR	78%^e^	N/A
Engelen, Bundy, Lau et al (2015)^e^ [51]	10% (accel): 1 misplaced, 1 NR	NR	5%, up to 20% (accelerometer)	NR	N/A	75%^e^
SASCHA B1 (2020) [48,50,62]	NR	NR	NR	NR	71%^e^	N/A
FLUX (2013) [38,52-57]	4%: NR. 17%^f^ (accel): 13 NR, 5 lost, 1 stolen	0%-1%: no paired data. 9% (accel): <4 days wear	2% (accelerometer)	12.5 (10-15)	66%^e^	N/A
Engelen, Bundy, Bauman et al (2015)^f^ [58]	2%: includes 1 school absence	13%^f^: “nonvalid data”	NR	NR	N/A	51%
Norman et al (2020) [32]	NR	10%: late daily surveys	NR	NR	N/A	49%^e^
Rovane et al (2024) [59]	NR	NR	NR	NR	N/A	NR
Family Matters B2 (2025) [60]	NR	31%^f^: did not “complete” study days	NR	NR	N/A	NR

a Values are the average time in minutes, and values within parentheses are the range, if reported.

b RR: response rate.

c NR: not reported.

d Not applicable.

e Calculated from provided study information (Multimedia Appendix 1).

f Protocols with >11% dropout were considered significant [22].

### Quantitative Data

Exclusion from analysis was the most commonly reported source of attrition (n=8), with criteria including incomplete daily surveys [41-47], nonvalid data exclusions [51], and fewer than 5 successive ratings [37]. Dropout rates ranged from 2% to 30% (n=4) [35,38-40,51-58]. Technical issues contributed to attrition in 4 protocols [35,36,38,51-57].

Fifteen protocols reported (n=6) or enabled calculation of a response rate (n=9), defined as the percentage of planned prompts completed. Six met the high-adherence threshold (≥80%) [33,36-47] and 10 fell below it (48%‐78%). Pooled estimates could be calculated only for 4 protocols using a random-effects model (mean 64.6%, 95% CI 53.6%‐75.5%), reflecting incomplete and inconsistent reporting ( Multimedia Appendix 4). Only Moschko et al [35] collected both child and parent data (62% child and 56% parent), limiting direct comparisons between self- and proxy report. Five protocols reported response latency, the time between prompt delivery and participant response, averaging 6 minutes [31,35,38-47,52-57].

### Qualitative Data

Only 2 of the 17 protocols [31,32] incorporated formal qualitative interviews, one using manifest analysis of parent interviews [32] and the other summarizing joint child-parent interviews [31], so we supplemented these findings with extracted observations and author reflections from the “Results” and “Discussion” sections of the remaining 13 protocols. Using the work of Norman et al [32] as a foundation, we identified 4 facilitators and 6 barriers (Tables5  6).

**Table 5.** Facilitators to acceptability and feasibility reported across protocols (research question 2).

Theme	Details
Uncomplicated (n=3) [31-33]	EMA^a^ tools were easy to use or included simplifying features. Familiar tasks (eg, taking photographs) required little explanation, prompts included clear training and visuals, and participation was described as not a major inconvenience.
Engaging (n=2) [31,32]	Participants found EMA tasks enjoyable and reported positive attitudes, with reference to educational benefits for children.
Caregiver support (n=1) [31]	Parents helped children interpret ambiguous prompts, families supported participation by listening for alerts when devices were left in shared spaces and reminding children to respond, and school staff enabled access and ensured audible alerts during school hours.
Reminders (n=3) [31,32,41]	Ongoing researcher contact supported engagement. Norman et al [32] added an end-of-day survey mid-study to address low event-contingent response rates, which was reflected on positively.

a EMA: ecological momentary assessment.

**Table 6.** Barriers to acceptability and feasibility reported across protocols (research question 2).

Theme	Details
Demanding (n=3) [32,61]	EMA^a^ prompts were seen as burdensome due to high effort or intensity, which was linked to dropout; timing (eg, during busy mornings) also contributed to perceived burden.
Challenging to report accurately (n=6) [31,32,47,54,61]	Both child self-reporters and proxy reporters struggled with precise reporting. Children exhibited extreme response bias on Likert or VAS^b^ scales and often could not map prompts onto real-world activities, sometimes needing caregiver help, while proxies found it difficult to categorize nonconforming events (eg, buffet-style meals) and potentially skewed responses due to social desirability or guessing when not observing the child.
Device awareness (n=3) [31,32,38,52-57]	Devices were frequently forgotten, left uncharged, or misplaced. Norman et al [32] found that proxy-reporting parents often overlooked event-contingent prompts, compromising data completeness.
Device access (n=3) [[31,36]	Children sometimes lost physical access to devices due to extracurricular commitments, restrictive school policies, examinations, or institutional rules; caregiver control (eg, muting or storing devices) further limited engagement.
Stigma (n=1) [31]	Using EMA devices in school led to peer questioning and discomfort. In one instance, stigma resulted in a participant discontinuing EMA participation during school hours.
Lack of caregiver support (n=2) [31,36]	Participation decreased when caregivers lacked time to assist children or when teaching staff required children to mute or silence devices during lessons.

a EMA: ecological momentary assessment.

b VAS: visual analog scale.

We introduce a third category, mitigators, based on participant and author suggestions for addressing barriers. Only Vilaysack et al [31] reported participant feedback, which included improving notification sounds and providing a carrying case for the EMA collection device. The authors agreed with these suggestions, recommending louder, longer alerts and training children to adjust volume settings or automating them to suit school environments [31]. A carrying bag was also recommended for future use [31].

Three protocols recommended including additional reporters including the child (n=1) [37], primary caregivers (n=1) [38,52-57], secondary caregivers (n=1) [41-47], and teachers (n=1) [37]. Alternative strategies to improve data collection, particularly in reference to children’s difficulties expressing their experiences, included combining EMA with qualitative methods (n=3) [37,41-47,60]; using sensor data to add contextual information (n=3) [35,38,52-57,60]; and extending the monitoring period to account for inconsistencies in responses (n=3) [41-47,51,58]. Authors also emphasized the need for training children and parents to express experiences effectively, particularly in mapping experiences to scales (n=1) [31], and acquiring subject knowledge (n=1) [41-47]. Two protocols recommended greater customization, including adapting data collection for participants whose first language differed from that of the researchers or protocol [41-47], offering optional custom reminders [58].

#### *Research question 3*: What characteristics of the EMA protocol (RQ1) are related to high and low response rate (RQ2) when using EMA with children between the ages of 5 and 11 years?

Of the protocols with a reported or calculated response rate (n=15/17), high- and low response rate characteristics are summarized in Table 7. However, as the majority of protocols were judged at critical risk of bias, these comparisons should be interpreted with caution. High response rate protocols (n=6) [33,36-47] predominantly recruited older KS2 children with ADHD and relied on parent report. In contrast, low response rate protocols (n=9) [31,32,35,38,40,48-58,62] were evenly split between KS1 and KS2, typically included children without identified health conditions, and more commonly used child self-report. Both groups used verbal instruction, but hands-on practice and written instruction were more common in low-response protocols. Both groups also used handheld devices, although smartphones specifically were more common in low response rate protocols [33-35,61,63,64]. Monitoring periods differed, with longer durations in high-response protocols (≥3 weeks) and shorter durations (<3 weeks) in low-response protocols.

**Table 7.** Comparison of high and low response rate ecological momentary assessment protocols with children aged 5-11 years, showing majority design features within each group.

Characteristic	High response rate	Low response rate
Demographics	KS1^a^ children (n=1/6), KS2^b^ children (n=5/6)	KS1 children (n=5/9), KS2 children (n=5/9)
	ADHD^c^ (n=4/6)	No condition (n=8/9)
	Children reporting (n=2/6) Parents reporting (n=5/6)	Children reporting (n=5/9) Parents reporting (n=5/9)
Training	Verbal instruction (n=4/6) Written instruction (n=1/6) Practice for reporter (n=3/6)	Verbal instruction (n=6/9) Written instruction (n=4/9) Practice for reporter (n=6/9)
Technology	Smartphone (n=2/6)	Smartphone (n=7/9)
Monitoring period	≥3 weeks (n=4/6)	≥3 weeks (n=3/9)
	<3 weeks (n=2/6)	<3 weeks (n=6/9)
Prompt frequency	3 prompts per day (n=4/6)	3-4 prompts per day (n=6/9)
Prompting design	Interval-contingent (n=6/6) Random (n=1/6) Fixed (n=5/6)	Interval-contingent (n=9/9) Random (n=5/9) Fixed (n=2/9)
	Prompts before school, after school, and evening (n=5/6)	Prompts before school, after school, and evening (n=2/9)
	≤12 items per prompt (n=2/6)	≤12 items per prompt (n=6/9)
	>13 items per prompt (n=4/6)	>13 items per prompt (n=3/9)
	Use of scales for responses (n=6/6)	Use of scales for responses (n=7/9)
	Multiple response types in 1 prompt (n=3/6)	Multiple response types in 1 prompt (n=7/9)
	≤1 hour to respond to prompt (n=1/6)	≤1 hour to respond to prompt (n=6/9)
Design features	Monetary reward (n=4/6)	Monetary reward (n=5/9)
	Prompt timing customization (n=6/6)	Prompt timing customization (n=3/6)

a KS1: key stage 1.

b KS2: key stage 2.

c ADHD: attention-deficit/hyperactivity disorder.

All protocols used interval-contingent sampling with 3-4 prompts each day, and high response rate protocols predominantly used fixed schedules, typically avoiding school hours (n=4/6) by prompting before school, after school, and in the evening. In contrast, low response rate protocols used random schedules. Based on the maximum number of items used in a single prompt within each protocol, high-response protocols typically included 20 or more items (n=4/6), while low-response protocols more often included 12 or fewer items (n=5/8). Both groups used scale-based data collection methods, but low-response protocols more frequently combined multiple response formats within a single prompt and imposed shorter response windows of 1 hour or less. In the high response rate group, most protocols offered monetary incentives and all allowed participants to customize prompt timing; by contrast, in the low-response group, incentives were less consistently used and prompt timing customization was uncommon.

## Discussion

### Principal Findings

This review aimed to answer 3 RQs by identifying the characteristics of EMA protocols used with children aged 5-11 years (RQ1), the feasibility and acceptability of EMA protocols for children aged 5-11 years (RQ2), and the protocol characteristics linked to high and low response rates (RQ3). Most protocols used handheld devices, interval-contingent prompting, and Likert scales, with training usually verbal and practice-based to collect self- or parent-reported data across monitoring periods of 3-28 days (RQ1). Feasibility and acceptability remain difficult to assess due to heterogeneous reporting and that 13 of 17 protocols were rated at critical risk of bias using the ROBINS-I tool. Key data required for pooling (eg, raw counts for planned vs completed prompts and corresponding variance estimates) were missing or selectively reported, preventing meaningful quantitative synthesis. Reported facilitators of acceptability and feasibility included the protocol being uncomplicated and engaging, the use of reminders, and caregiver support. Barriers included device access issues, reporter difficulty reporting accurately, reporting burden, stigma, lack of protocol awareness, and insufficient caregiver involvement. Suggested future mitigations included improvements to reminder systems, carrying aids, longer monitoring periods, increased customization, passive data collection, and involving additional reporters or methods (RQ2). High-response protocols (n=6, ≥80%) [20] more often involved older children (KS2), those with specific health conditions (ADHD), and featured longer monitoring periods (≥3 weeks). These protocols typically used fixed, interval-contingent prompt schedules, with greater than 20 items and prompting 3 times a day, often before school, after school, and in the evening. The majority also offered limited customization of prompt timing and provided monetary reimbursement for participation. Given the high risk of bias across most protocols, these contrasts should be interpreted as tentative (RQ3).

### Comparison With Prior Work

Evidence on the feasibility and acceptability of EMA protocols for preadolescent children remains limited, and how EMA protocol design can best support these outcomes is unclear [22-26]. Despite this, there is a growing recognition that protocols should be adapted for this age group [23,24,26]. To our knowledge, this is the first review to systematically examine the feasibility and acceptability of EMA protocols for preadolescent children. While challenges such as technical issues and participant burden are common across age groups [1,19,65], this review identified additional factors that appear especially important for preadolescents and their caregivers acting as proxy reporters, including difficulties responding to prompts accurately, inconsistent access to reporting devices, and device-related stigma. To explore the impact of protocol characteristics on acceptability and feasibility, we apply the Technology Acceptance Model, which identifies perceived ease of use and perceived usefulness as key influences on technology adoption [66]. Using this framework helps distinguish between protocol features that may have supported acceptability, those that produced unexpected patterns, and those that created barriers to technology acceptability.

Several features of high response rate protocols appeared to align with the Technology Acceptance Model’s concept of perceived ease of use and usefulness, potentially making participation more acceptable. Predictable prompting schedules (eg, before school, after school, and evening), opportunities to customize timing, and the avoidance of in-school prompts may have helped reduce disruption (eg, limiting negative teacher interference) and make participation feel easier to manage within family routines [31,67]. The use of familiar technologies to caregivers, uncomplicated interfaces, encouraging caregiver support in responding to prompts and simple visuals may have supported participation by reducing cognitive and knowledge barriers, thereby improving perceived ease of use [68]. Perceived usefulness may have been reinforced through external motivators such as monetary incentives and through the involvement of caregivers, including school staff, who facilitated tasks [69-72], and the framing of EMA tasks as enjoyable or educational [73].

Other features associated with high response rates, however, diverged from common EMA design practices and therefore require more cautious interpretation. Adult EMA literature typically favors shorter monitoring periods (≤2 weeks) [23,25] and brief prompts to reduce participant burden [16]. In contrast, in this review, longer periods (≥3 weeks) and extensive prompts (≥20 items per prompt) were present in the majority of high response rate protocols. One explanation may be that longer durations help embed EMA into daily routines, with the stability offsetting potential fatigue [1,22]. However, these more demanding protocols also tended to provide stronger external supports, such as closer researcher involvement or monetary incentives, suggesting that response rates may have reflected a combination of routine and additional resources rather than duration or length alone. Training showed a similar counterintuitive pattern: hands-on practice was more common in low response rate protocols, indicating that practice is more important for supporting valid and accurate reporting than for increasing response rates [74]. A further unexpected pattern was the higher response rates linked to children with ADHD. Although this might initially appear counterintuitive, children with long-term conditions may be more intrinsically motivated to engage, as they are more likely to recognize a personal need that technology could help address [75]. In contrast, children without a long-term health condition may not perceive the same relevance or potential benefit.

By contrast, several protocol features reduced perceived ease of use, reflecting both design complexity and developmental challenges. For example, nearly all protocols relied on response formats such as Likert or visual analog scales, despite evidence that alternative formats, such as item ranking and semantic differential scales, may be more developmentally appropriate for younger children [76-78]. The continued reliance on such scales highlights a broader adult-centered design approach that may overlook children’s cognitive abilities and real-world reporting capacities [79]. Furthermore, many 5‐7 year olds may struggle with reading, reinforcing the need for adult support in interpreting prompts [80,81]. Yet, reliance on caregivers may overlook the developmental trajectory of literacy: children who already have the skills may be denied opportunities to practice reporting independently, while those still acquiring literacy may be excluded altogether.

Beyond developmental challenges, logistical and practical barriers also reduced ease of use. More complex protocols, such as those combining multiple response formats or enforcing narrow response windows (<1 hour), may have placed additional cognitive and logistical demands on children and caregivers [82,83]. These challenges are especially important, given the documented difficulty, in both this review and wider literature, that children face with extreme response bias and mapping their experiences onto structured input formats [80]. Furthermore, the lack of flexibility beyond initial prompt timing customization contrasts with adult EMA, where participants can sometimes delay prompts or choose alternative response formats to reduce burden [84,85]. For example, 1 protocol using photo-based meal tracking struggled to capture buffet-style eating, illustrating how rigid input formats can fail to reflect real-world variability [32]. While this is a known limitation for adults [1], it may be amplified in children, as caregivers may resist disrupting routines and children may see little benefit in adjusting their behavior to enable easier data capture [67,86]. Additionally, lack of device awareness contributed to issues such as forgetting to charge it or bring it with them, disrupting participation [23]. These challenges may be greater for preadolescents, who may not own devices suitable for EMA and are instead given separate ones, making routine integration more difficult [23,30]. Using wearables, which remain on the body, may help reduce this burden [16,87]; however, no protocol in this review used such an approach. Three protocols recommended incorporating sensor data to contextualize and support children’s self-reports, suggesting a potential role for wearables in future protocols [88].

Perceived usefulness was often less prioritized than ease of use. While many protocols included monetary incentives, the included protocols did not report how they established personal relevance for either children or caregivers. Emphasizing the value of participation may be particularly important when EMA is used solely as a measurement tool, as was the case in all included protocols, to avoid participants feeling that they contribute data without clear benefits or autonomy [8,89]. In addition, preadolescent children may be better motivated by immediate rewards [90], yet only 1 protocol in this review included a daily reward system [41-47]. Social stigma also shaped perceived usefulness. In 1 protocol, preadolescents reported attracting unwanted attention when using a smartphone at school [31]. This highlights how adult assumptions about appropriate technologies can overlook that many preadolescents lack regular access to smartphones [91,92], which may contribute to feelings of unfamiliarity or stigma. Few protocols in this review involved children directly in their development (eg, piloting), limiting opportunities to identify usability issues and integrate children’s perspectives [93,94].

Preadolescents are embedded in a shifting network of caregivers, teachers, peers, and wider family [95], yet many protocols included in this review treat them as isolated participants and do not explicitly involve caregivers in supporting roles. While caregivers were often given verbal or written instructions when the child was the reporter, protocols rarely included opportunities for them to practice using the device. School cooperation was also limited, restricting access to essential adult support. Furthermore, simultaneous tracking by children and caregivers was rare, although others recommended incorporating multiple perspectives in future work [37,38,41-47,52-57]. Such approaches may offer benefits including shared responsibility, enhanced motivation, and enriched data perspectives [96,97]. Preadolescents rely heavily on adults and their peers to interpret, manage, and respond to daily tasks [98,99], making limited involvement of these networks in protocol design both a missed opportunity and a barrier to engagement [31]. Protocols that acknowledge and actively involve this network, through co-design, piloting, and contextual adaptation, are more likely to avoid common pitfalls and create experiences that are not only feasible but also genuinely valuable to children and those who support them [100-102], although this must be balanced against the risk that proxy reporting shifts the focus away from children’s voices. In KS1 protocols, for example, parents often acted as reporters, sometimes entering responses in dialogue with their child, but in other cases responding based only on their own observations. The latter approach arguably reflects EMA of parents about their children, rather than EMA of children themselves, raising questions of validity and comparability with adult EMA where self-report is standard. These issues highlight the importance and complexity of situating EMA within children’s lives, emphasizing the need for EMA protocols that fit within children’s everyday social and caregiving contexts, ensuring that both children and their caregivers can meaningfully engage with and benefit from these tools [70,103].

### Strengths and Limitations

Digital health is an interdisciplinary field, and a key strength of this review is the interdisciplinary approach taken by searching a large number of databases (n=10), spanning both health science databases and HCI venues. The review aimed to investigate EMA protocols used with children (aged 5‐11 years) to understand developmentally sensitive design implications. To investigate this, we looked across the broad spectrum of child health behaviors and conditions, which means our review is not limited to any specific health domain, making the findings relevant to a broader scope of researchers working with pediatric EMA protocols. However, due to heterogeneity and lack of reporting, a formal meta-analysis of quantitative data nor metaethnography of qualitative data was feasible, and we instead used a narrative review approach [61] to identify patterns that may inform future hypothesis generation. We also excluded studies that included our target age group but did not report specific protocol adaptations for younger children (eg, studies covering ages 5‐18 years without age-specific design considerations). The use of narrative synthesis, while appropriate given the diversity of study design, has been critiqued for limited transparency [104]. Additionally, the scarcity of detailed qualitative and quantitative data restricted the depth of our analysis. This limitation highlights a need for future research exploring child and caregiver views to improve EMA protocols. A further limitation is that most protocols were conducted in Western, high-income contexts, with little reporting of socioeconomic background and inconsistent reporting of ethnicity. Where provided, samples were often predominantly White, with only a few protocols including more diverse populations. Future research should prioritize more diverse samples and clearer demographic reporting.

### Conclusions

This review provides the first systematic evidence base focused exclusively on digital EMA with preadolescent children. It examined 17 digital EMA protocols involving children aged 5‐11 years, highlighting gaps in developmental appropriateness (eg, absence of child-focused piloting) and inconsistencies in reporting quality that limit both interpretability and comparability across studies.

In contrast to existing reviews that primarily emphasize adherence or feasibility in children of different ages as a single group and adults, these findings emphasize the importance of preadolescent acceptance and developmental considerations when EMA is used with this specific age group. Barriers included device access issues, finding it challenging to report accurately (eg, response options not matching how they want to express themselves), reporting burden, stigma, lack of protocol awareness, and insufficient caregiver involvement. Facilitators included uncomplicated, engaging technology, reminders, and caregiver involvement. Several counterintuitive patterns also emerged: protocols with longer durations and more items per prompt were linked to high response rates, further highlighting the importance of considering preadolescents distinctly.

The review contributes to the field by consolidating the evidence base and identifying protocol characteristics, while also highlighting the need for improved and more consistent reporting of feasibility, acceptability, and response metrics. Without such improvements, meaningful comparison across protocols and cumulative knowledge building remains limited.

From a practical perspective, the findings suggest that future digital EMA research should focus on perceived ease of use (eg, predictable prompting schedules, simplified response formats, and flexibility in fitting daily routines) and perceived usefulness (eg, immediate rewards, personally relevant activities, clear explanations of purpose, and addressing stigma) as part of a more child-centered design approach. Given children’s dependence on caregivers and teachers, involving these adults is likely to support perceived ease of use (eg, assistance with prompts, device availability, and charging) and perceived usefulness (eg, clarifying relevance, reinforcing engagement, and managing social dynamics). With greater developmental alignment and improved reporting standards, digital EMA could be more effectively integrated into pediatric health monitoring in ways that are sensitive to the needs of different age groups.

## Supplementary material

10.2196/79291 Multimedia Appendix 1 Full search strategy.

10.2196/79291 Multimedia Appendix 2 Quality assessment including CREMAS (Checklist for Reporting EMA Studies) and ROBINS-I v2 (Risk Of Bias In Nonrandomized Studies of Interventions, version 2).

10.2196/79291 Multimedia Appendix 3 Study-level overviews.

10.2196/79291 Multimedia Appendix 4 Response rate calculations.

10.2196/79291 Multimedia Appendix 5 Data extraction tables (full).

10.2196/79291 Checklist 1 PRISMA (Preferred Reporting Items for Systematic Reviews and Meta-Analyses) checklist.

10.2196/79291 Checklist 2 PRISMA-S (An Extension to the PRISMA Statement for Reporting Literature Searches in Systematic Reviews) checklist.

## Abbreviations

ADHD: attention deficit/hyperactivity disorder
ASD: autism spectrum disorder
CREMAS: Checklist for Reporting EMA Studies
EMA: ecological momentary assessment
HCI: Human-Computer Interaction
KS1: key stage 1
KS2: key stage 2
PRISMA: Preferred Reporting Items for Systematic Reviews and Meta-Analyses
ROBINS-I v2: Risk Of Bias In Nonrandomized Studies of Interventions, version 2
RQ: research question
