# Supplementary material for: A Health-Related Digital Ecological Momentary Assessment in Children (Aged 5– 11 Years): Systematic Review
Source: J Med Internet Res. 2026 Apr 14;28:e79291. doi: 10.2196/79291 (PMC13078612; doi:10.2196/79291)
Supplement: Multimedia Appendix 1 [file jmir-v28-e79291-s001.docx]

**Multimedia Appendix 1. Search Strategies**

The literature search was first conducted in on the 13th March 2023 and updated in on the 28th October 2024 and 15th October 2025 in the following databases: CINAHL, ACM Digital Library, PsycINFO, Embase, MEDLINE, Cochrane Library, IEEE Xplore, PubMed, Scopus, and Web of Science.

Ecological momentary assessment terms were based on terms of Yang et al [28].

Child terms were based on MeSH terms created by Boluyt et al [29].

**Search Strategy for Medline via Ovid**

1 (child* or Paediatric* or Pediatric* or Juvenil* or "Minor" or "Minors" or "School child" or "school children" or Schoolchild* or "Kid" or "kids" or pre-adolescen* or preadolescen* or "Primary school child" or "primary school children" or "elementary school child" or "elementary school children" or "middle school child" or "middle school children" or "Junior school child" or "junior school children" or Pre-teen* or preteen* or pubescen* or pre-pubescen* or prepubescen* or "boy" or "boys" or "girl" or "girls" or stepchild* or step-child* or (young* adj3 child*)).ti,ab,kf.

2 ("Ecological Momentary Assessment" or "EMA" or "Experience Sampling" or "ESM" or "Daily Diary Approach" or "Ambulatory Assessment" or "Event Sampling" or "Beeper Studies" or "Structured Diary Method" or "Intensive Longitudinal Assessment" or "Real-Time Data Capture").ti,ab,kf.

3 ("epithelial membrane antigen" or ("EMA" adj3 "licensed") or ("EMA" adj3 "approved") or "European Medicines Agency").ti,ab,kf

4 (1 and 2) not 3

**Search Strategy for PhycInfo via Ovid**

1 (child* or Paediatric* or Pediatric* or Juvenil* or "Minor" or "Minors" or "School child" or "school children" or Schoolchild* or "Kid" or "kids" or pre-adolescen* or preadolescen* or "Primary school child" or "primary school children" or "elementary school child" or "elementary school children" or "middle school child" or "middle school children" or "Junior school child" or "junior school children" or Pre-teen* or preteen* or pubescen* or pre-pubescen* or prepubescen* or "boy" or "boys" or "girl" or "girls" or stepchild* or step-child* or (young* adj3 child*)).tw

2 ("Ecological Momentary Assessment" or "EMA" or "Experience Sampling" or "ESM" or "Daily Diary Approach" or "Ambulatory Assessment" or "Event Sampling" or "Beeper Studies" or "Structured Diary Method" or "Intensive Longitudinal Assessment" or "Real-Time Data Capture").tw.

3 "

(""epithelial membrane antigen"" or (""EMA"" adj3 ""licensed"") or (""EMA"" adj3 ""approved"") or ""European Medicines Agency"").tw."

4 (1 and 2) not 3

**Search Strategy for PubMed via PubMed**

(child* or Paediatric* or Pediatric* or Juvenil* or "Minor" or "Minors" or "School child" or "school children" or Schoolchild* or "Kid" or "kids" or pre-adolescen* or preadolescen* or "Primary school child" or "primary school children" or "elementary school child" or "elementary school children" or "middle school child" or "middle school children" or "Junior school child" or "junior school children" or Pre-teen* or preteen* or pubescen* or pre-pubescen* or prepubescen* or "boy" or "boys" or "girl" or "girls" or stepchild* or step-child* or "young child"[Title/Abstract:~3]) AND ("Ecological Momentary Assessment" or "EMA" or "Experience Sampling" or "ESM" or "Daily Diary Approach" or "Ambulatory Assessment" or "Event Sampling" or "Beeper Studies" or "Structured Diary Method" or "Intensive Longitudinal Assessment" or "Real-Time Data Capture") NOT ("epithelial membrane antigen" or "EMA licensed"[Title/Abstract:~3] or "EMA approved"[Title/Abstract:~3] or "European Medicines Agency")

**Search Strategy for Scopus**

( ( TITLE-ABS-KEY ( child* OR paediatric* OR pediatric* OR juvenil* OR {Minor} OR {Minors} OR {School child} OR {school children} OR schoolchild* OR {Kid} OR {kids} OR pre-adolescen* OR preadolescen* OR {Primary school child} OR {Primary school children} OR {elementary school child} OR {elementary school children} OR {middle school child} OR {middle school children} OR {Junior school child} OR {Junior School children} OR pre-teen* OR preteen* OR pubescen* OR pre-pubescen* OR prepubescen* OR {boy} OR {boys} OR {girl} OR {girls} OR stepchild* OR step-child* OR ( young* W/3 child* ) ) ) ) AND ( TITLE-ABS-KEY ( {Ecological Momentary Assessment} OR {EMA} OR {Experience Sampling} OR {ESM} OR {Daily Diary Approach} OR {Ambulatory Assessment} OR {Event Sampling} OR {Beeper Studies} OR {Structured Diary Method} OR {Intensive Longitudinal Assessment} OR {Real-Time Data Capture} ) ) AND NOT ( TITLE-ABS-KEY ( {epithelial membrane antigen} OR ( ema W/3 licensed ) OR ( ema W/3 approved ) OR {European Medicines Agency} ) )

**Search Strategy for Web of Science via Web of Science**

1 (child* or Paediatric* or Pediatric* or Juvenil* or "Minor" or "Minors" or "School child" or "school children" or Schoolchild* or "Kid" or "kids" or pre-adolescen* or preadolescen* or "Primary school child" or "primary school children" or "elementary school child" or "elementary school children" or "middle school child" or "middle school children" or "Junior school child" or "junior school children" or Pre-teen* or preteen* or pubescen* or pre-pubescen* or prepubescen* or "boy" or "boys" or "girl" or "girls" or stepchild* or step-child*)

2 "Ecological Momentary Assessment" or "EMA" or "Experience Sampling" or "ESM" or "Daily Diary Approach" or "Ambulatory Assessment" or "Event Sampling" or "Beeper Studies" or "Structured Diary Method" or "Intensive Longitudinal Assessment" or "Real-Time Data Capture"

3 ("epithelial membrane antigen" or "European Medicines Agency")

**Search Strategy for Embase via Embase**

1 (child* or Paediatric* or Pediatric* or Juvenil* or "Minor" or "Minors" or "School child" or "school children" or Schoolchild* or "Kid" or "kids" or pre-adolescen* or preadolescen* or "Primary school child" or "primary school children" or "elementary school child" or "elementary school children" or "middle school child" or "middle school children" or "Junior school child" or "junior school children" or Pre-teen* or preteen* or pubescen* or pre-pubescen* or prepubescen* or "boy" or "boys" or "girl" or "girls" or stepchild* or step-child* or (young* adj3 child*)).ti,ab,kf.

2 ("Ecological Momentary Assessment" or "EMA" or "Experience Sampling" or "ESM" or "Daily Diary Approach" or "Ambulatory Assessment" or "Event Sampling" or "Beeper Studies" or "Structured Diary Method" or "Intensive Longitudinal Assessment" or "Real-Time Data Capture").ti,ab,kf.

3 ("epithelial membrane antigen" or ("EMA" adj3 "licensed") or ("EMA" adj3 "approved") or "European Medicines Agency").ti,ab,kf

4 (1 and 2) not 3

**Search Strategy for CINHAL via CINHAL**

1 (child* or Paediatric* or Pediatric* or Juvenil* or "Minor" or "Minors" or "School child" or "school children" or Schoolchild* or "Kid" or "kids" or pre-adolescen* or preadolescen* or "Primary school child" or "primary school children" or "elementary school child" or "elementary school children" or "middle school child" or "middle school children" or "Junior school child" or "junior school children" or Pre-teen* or preteen* or pubescen* or pre-pubescen* or prepubescen* or "boy" or "boys" or "girl" or "girls" or stepchild* or step-child* or (young* N3 child*))

2 ("Ecological Momentary Assessment" or "EMA" or "Experience Sampling" or "ESM" or "Daily Diary Approach" or "Ambulatory Assessment" or "Event Sampling" or "Beeper Studies" or "Structured Diary Method" or "Intensive Longitudinal Assessment" or "Real-Time Data Capture")

3 ("epithelial membrane antigen" or ("EMA" N3"licensed") or ("EMA" N3 "approved") or "European Medicines Agency")

**Search Strategy for Cochrane via Cochrane**

1 (child* or Paediatric* or Pediatric* or Juvenil* or "Minor" or "Minors" or "School child" or "school children" or Schoolchild* or "Kid" or "kids" or pre-adolescen* or preadolescen* or "Primary school child" or "primary school children" or "elementary school child" or "elementary school children" or "middle school child" or "middle school children" or "Junior school child" or "junior school children" or Pre-teen* or preteen* or pubescen* or pre-pubescen* or prepubescen* or "boy" or "boys" or "girl" or "girls" or stepchild* or step-child* or (young* adj3 child*))

2 ("Ecological Momentary Assessment" or "EMA" or "Experience Sampling" or "ESM" or "Daily Diary Approach" or "Ambulatory Assessment" or "Event Sampling" or "Beeper Studies" or "Structured Diary Method" or "Intensive Longitudinal Assessment" or "Real-Time Data Capture")

3 ("epithelial membrane antigen" or ("EMA" adj3 "licensed") or ("EMA" adj3 "approved") or "European Medicines Agency")

4 #1 AND #2 NOT #3

**Search Strategy for ACM via ACM**

[[All: child*] OR [All: paediatric*] OR [All: pediatric*] OR [All: juvenil*] OR [All: "minor"] OR [All: "minors"] OR [All: "school child"] OR [All: "school children"] OR [All: schoolchild*] OR [All: "kid"] OR [All: "kids"] OR [All: pre-adolescen*] OR [All: preadolescen*] OR [All: "primary school child"] OR [All: "primary school children"] OR [All: "elementary school child"] OR [All: "elementary school children"] OR [All: "middle school child"] OR [All: "middle school children"] OR [All: "junior school child"] OR [All: "junior school children"] OR [All: pre-teen*] OR [All: preteen*] OR [All: pubescen*] OR [All: pre-pubescen*] OR [All: prepubescen*] OR [All: "boy"] OR [All: "boys"] OR [All: "girl"] OR [All: "girls"] OR [All: stepchild*] OR [All: step-child*]] AND [[All: and] OR [All: "ecological momentary assessment"] OR [All: "ema"] OR [All: "experience sampling"] OR [All: "esm"] OR [All: "daily diary approach"] OR [All: "ambulatory assessment"] OR [All: "event sampling"] OR [All: "beeper studies"] OR [All: "structured diary method"] OR [All: "intensive longitudinal assessment"] OR [All: "real-time data capture"]] AND NOT [[All: "epithelial membrane antigen"] OR [All: "european medicines agency"]]

**Search Strategy for IEEE via IEEE**

child* OR Paediatric OR Pediatric OR Juvenile OR Minor OR "School child" OR "school children" OR Schoolchild* OR Kid OR pre-adolescen* OR preadolescen* OR pre-teen* OR preteen* OR pubescen* OR pre-pubescent OR prepubescent OR boy OR girl

AND

"Ecological Momentary Assessment" OR "EMA" OR "Experience Sampling" OR "ESM" OR "Daily Diary Approach" OR "Ambulatory Assessment" OR "Event Sampling" OR "Beeper Studies" OR "Structured Diary Method" OR "Intensive Longitudinal Assessment" OR "Real-Time Data Capture"
