# Supplementary material for: A Health-Related Digital Ecological Momentary Assessment in Children (Aged 5– 11 Years): Systematic Review
Source: J Med Internet Res. 2026 Apr 14;28:e79291. doi: 10.2196/79291 (PMC13078612; doi:10.2196/79291)
Supplement: Multimedia Appendix 3 [file jmir-v28-e79291-s003.docx]

**Multimedia Appendix 3. Study-level overviews.**

| Protocol | Study | Location | Age range (y) | Female | No. | Aim |
| --- | --- | --- | --- | --- | --- | --- |
| Family Matters | Trofholz et al (2021) [41] | US | 5-7 | NR | 150 families | Summarizes the development of the Family Matters study, which aimed to better understand diets of low-income, racially and ethnically diverse families. |
| Family Matters | Tate et al (2020) [42] | US | 5-7 | NR | 128 families (sub-sample) | Examining the relationship between daily stress and family dietary choices, including capturing child health-behaviors |
| Family Matters | Berge et al (2020) [43] | US | 5-7 | NR | 150 families | Using EMA to understand the associations between parental stress, parent feeding practices, and child eating behaviors in the context of food insecurity |
| Family Matters | Loth et al (2020) [45] | US | 5-7 | 47.00% | 150 families | Examining contextual differences between children's snacks and meals and assess the impact of demographic factors on these differences. |
| Family Matters | Loth et al (2021) [44] | US | 5-7 | NR | 150 families | Comparison of parental 24-hour dietary recall and parental proxy-reported EMA surveys on children's food group intake. |
| Family Matters | Brito et al (2020) [46] | US | 5-7 | 47.00% | 140 families (sub-sample) | Examining the relationship between parent-reported and accelerometer-measured physical activity and sedentary time in children |
| Family Matters | Wirthlin et al (2020) [47] | US | 5-7 | 47.00% | 150 families | Investigating associations between parent and child physical activity and eating behaviors in diverse families |
| FLUX (Assessment of Cognitive Performance FLUctuations in the School ConteXt) | Könen et al (2015) [52] | Germany | 8-11 | 40% | 110 children | Investigating the relationship between the variations in sleep behavior (e.g. last nights sleep, daytime sleepiness) to working memory |
| FLUX (Assessment of Cognitive Performance FLUctuations in the School ConteXt) | Kühnhausen et al (2013) [54] | Germany | 8-11 | 40% | 82 children (sub sample) | Examining the influence of physical activity on affect and the feasibility of using accelerometers to measure PA |
| FLUX (Assessment of Cognitive Performance FLUctuations in the School ConteXt) | Könen et al (2016) [53] | Germany | 8-11 | 40% | 110 children | Investigating the association between sleep behaviors and affect in elementary school children |
| FLUX (Assessment of Cognitive Performance FLUctuations in the School ConteXt) | Dirk and Schmiedek (2017) [55] | Germany | 8-11 | 40% | 110 children | Understanding the relationship between children's working memory and the disturbances they perceive |
| FLUX (Assessment of Cognitive Performance FLUctuations in the School ConteXt) | Neubauer et al (2019) [56] | Germany | 8-11 | 40% | 110 children | Investigating the impact of affect state on working memory |
| FLUX (Assessment of Cognitive Performance FLUctuations in the School ConteXt) | Schmidt et al (2019) [57] | Germany | 8-11 | 40% | 110 children | Relationship between children's feelings of relatedness to peers at school and affect |
| FLUX (Assessment of Cognitive Performance FLUctuations in the School ConteXt) | Kramer et al (2021) [38] | Germany | 8-11 | 40% | 110 children | Exploring and validating ambulatory scales to measure rumination and worry, focusing on rumination |
| SASCHA B1 | Schmidt et al (2020) [50] | Germany | 9-11 | 54.00% | 90 children | Investigating fluctuations of self-esteem before the transition to secondary school |
| SASCHA B1 | Schmidt et al (2020) [50] | Germany | 9-11 | 54.00% | 90 children | Investigate the role of peer relatedness to wellbeing before transition to secondary school. |
| SASCHA B1 | Blume et al (2022) [48] | Germany | 9-11 | 54.00% | 90 children | Tracking academic success against day to day variations of self-regulation working memory and achievement goals before academic transition |
| SASCHA B2 | Schmidt et al (2020) [50] | Germany | 9-11 | 56% | 108 children | Investigating fluctuations of self-esteem after transitioning to secondary school |
| SASCHA B2 | Schmidt et al (2020) [50] | Germany | 9-11 | 56% | 108 children | Investigate the role of peer relatedness to wellbeing after transition to secondary school. |
| SASCHA B2 | Neubauer et al (2021) [40] | Germany | 9-11 | 56% | 108 children | Gain a greater understanding of the relationship between sleep behavior and affect wellbeing throughout the day. |
| SASCHA B2 | Galeano-Keiner et al (2022) [49] | Germany | 9-11 | 56.00% | 108 children | Exploring the link between morning and afternoon working memory and nightly sleep behavior. |
| SASCHA B2 | Blume et al (2022) [48] | Germany | 9-11 | 44% | 108 children | Tracking academic success against day to day variations of self-regulation working memory and achievement goals after academic transition |
| Jacobs Foundation | Kramer et al (2021) [38] | Germany | 8-10 | 44% | 84 children | Exploring and validating ambulatory scales to measure rumination and worry, focusing on worry |
| Jacobs Foundation | Neubauer et al (2021) [40] | Germany | 9-10 | 44% | 84 children | Gain a greater understanding of the relationship between sleep behavior and affect wellbeing throughout the day. |
| Jacobs Foundation | Neubauer et al (2022) [39] | Germany | 8-10 | 44% | 84 children | Validating ambulatory scale on need satisfaction and frustration. |
| The Sydney Playground Project | Engelen et al (2015) [58] | Australia | 5-7 | 46% | 246 dyads (parent or other caregiver) | Examining the feasibility of using EMA to describe patterns of after-school activity |
| The Sydney Playground Project | Engelen et al (2015) [51] | Australia | 5-7 | 45% | 20 dyads (parent or other caregiver) | Exploring the relationship between level of activity and contextual factors. |
| AttentionGO | Moschko et al (2022) [35] | Germany | 9-11 | 56% | 70 dyads | Investigating daily fluctuations in self-regulation among children with ADHD and their parent-child interactions. |
| None | Rosen and Epstein (2010) [33] | US | 8-9 | 0% | 2 dyads | Investigating the feasibility and utility of EMA to measure emotional dysregulation in children with ADHD and bipolar disorder. |
| None | Rosen et al (2013) [36] | US | 8-11 | 18% | 11 dyads | Exploring how parents can use EMA to proxy report on their child's emotional dysregulation. |
| None | Rosen et al (2013) [36] | US | 8-11 | 20% | 5 dyads (subsample) | Exploring how children can use EMA to self-report on their own emotional dysregulation. |
| None | Vilaysack et al (2016) [31] | Australia | 5-7 | 50% | 10 children | Examining the feasibility of the EMA method with typically developing children |
| None | Norman et al (2020) [32] | Sweden | 5-7 | 42% | 20 dyads | Examining the feasibility and validity of photograph based EMA of children's diets |
| None | Rovane et al (2025) [59] | US | 5-11 | 23% (1.1% NB) | 92 dyads | Exploring the relationship between parental emotional regulation, momentary parent stress and child behavior problems in children with ASD. |
| None | Alacha et al (2024) [37] | US | 7-11 | 40% | 47 dyads | Examining the influence of positive affect variability on homework problems in children with ADHD |
| Family Matters B2 | [60] | US | 5-7 | 54% | 436 dyads | Examining how parental stress, mood, and coping relate to children’s physical activity and screen-time in daily life. |
| None | Appelhans et al (2025) [34] | US | 5-10 | 57% | 60 dyads | Examining whether parent-supported recreational activities can displace discretionary eating and electronic entertainment. |
